# Diverse Mechanisms of Sulfur Decoration in Bacterial tRNA and Their Cellular Functions

**DOI:** 10.3390/biom7010033

**Published:** 2017-03-22

**Authors:** Chenkang Zheng, Katherine A. Black, Patricia C. Dos Santos

**Affiliations:** 1 Department of Chemistry, Wake Forest University, Winston-Salem, NC 27101, USA; zhenc13@wfu.edu; 2 Weill Cornell Medical College, New York City, NY 10065, USA

**Keywords:** tRNA, thionucleoside, 2-thiouridine, 4-thiouridine, 2-methylthioadenosine, cysteine desulfurases, thiouridylase, thiocofactor

## Abstract

Sulfur-containing transfer ribonucleic acids (tRNAs) are ubiquitous biomolecules found in all organisms that possess a variety of functions. For decades, their roles in processes such as translation, structural stability, and cellular protection have been elucidated and appreciated. These thionucleosides are found in all types of bacteria; however, their biosynthetic pathways are distinct among different groups of bacteria. Considering that many of the thio-tRNA biosynthetic enzymes are absent in Gram-positive bacteria, recent studies have addressed how sulfur trafficking is regulated in these prokaryotic species. Interestingly, a novel proposal has been given for interplay among thionucleosides and the biosynthesis of other thiocofactors, through participation of shared-enzyme intermediates, the functions of which are impacted by the availability of substrate as well as metabolic demand of thiocofactors. This review describes the occurrence of thio-modifications in bacterial tRNA and current methods for detection of these modifications that have enabled studies on the biosynthesis and functions of S-containing tRNA across bacteria. It provides insight into potential modes of regulation and potential evolutionary events responsible for divergence in sulfur metabolism among prokaryotes.

## 1. Introduction

**Figure 1:**
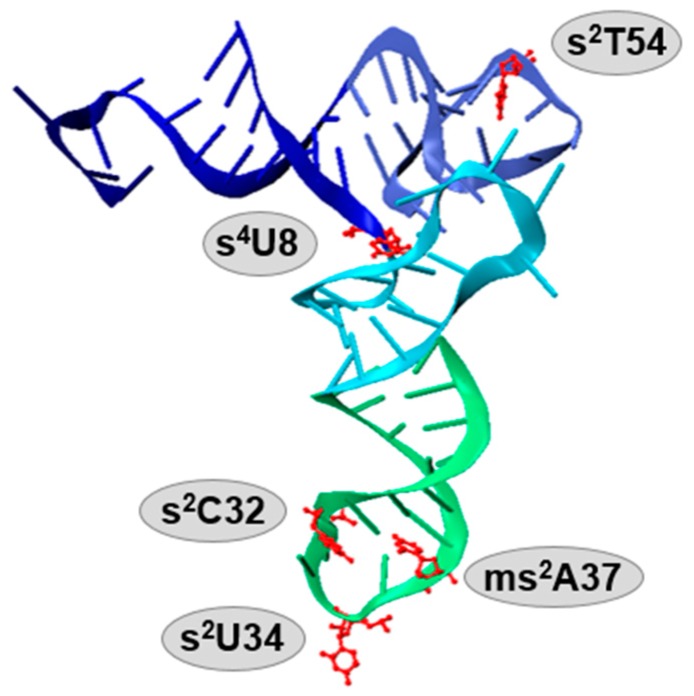
Location of thio-modifications in transfer ribonucleic acid (tRNA). The structure of a tRNA^Gln^ transcript (4jxz) with the positions of modified thionucleotides (red) was obtained in complex with *Escherichia coli* glutaminyl-tRNA synthetase (GlnRS) (not shown). The tRNA backbone and other nucleotides are colored by regions within which they are located on the tRNA as follows: The conserved CCA sequence to which amino acids are attached at the 3′ end is shown in navy, acceptor stem in royal blue, the D-loop in cyan, the anticodon loop in green, and the T-loop in periwinkle. It is important to note that in this isoacceptor, only positions 34 and 37 carry thionucleotides, and that this figure means to represent the locations of such modifications within a tRNA tertiary structure, rather than accurate information about a specific isoacceptor.

**Figure 2:**
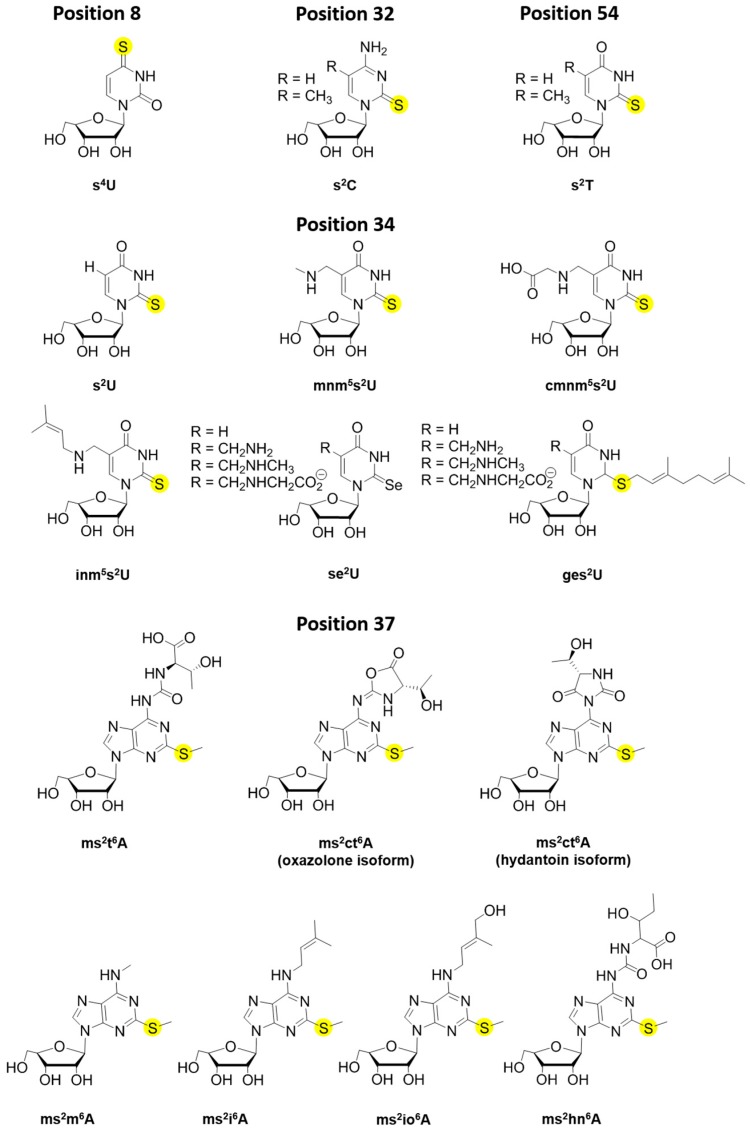
Structures of bacterial tRNA thionucleosides and their derivatives. The structures of the thionucleosides occurring in bacterial tRNA at positions 8, 32, 34, 37 and 54 are shown along with their hypermodified derivatives, with the sulfur atom highlighted in yellow. The structures of 2-geranylthiouridine (ges^2^U) and 2-selenouridine (se^2^U), whose biosynthesis relies on U34 thiolation, as described in Table 1, are also depicted along with their derivatives. mnm^5^s^2^U, cmnm^5^s^2^U, and inm^5^s^2^U may be methylated at 2′ OH. The cyclic form of t^6^A37 was first hypothesized to exist in the oxazolone isoform, however, further investigation has revealed that it actually exists in the hydantoin isoform.

Transfer ribonucleic acid (tRNA) is an essential facilitator in shuttling the genomic information between DNA and proteins. Nucleotide monomers consist of a ribose sugar, a phosphate group, and one of four different bases: adenine, uracil, cytosine, and guanine. These nucleotides can be post-transcriptionally modified to provide RNA with expanded chemical functionalities and afford diverse biological functions. Such chemical alterations have also been observed and studied mostly on ribosomal RNA (rRNA) and some messenger RNA (mRNA) [1,2,3]. More than 100 RNA modifications have been discovered thus far, and the majority are found within tRNA molecules [3,4,5,6]. Most of these “decorations” are derived from methylation, thiolation, and other more complex hypermodifications [7]. For example, pseudouridine (Ψ), a modification so abundant that it was identified as a “fifth nucleoside” in RNA, is an isomerization product resulting in a C–C rather than a N–C glycosyl bond of uridine which links between the base and sugar [8]. Nucleoside thiolation is another widely occurring modification (Figure 1), including products such as 4-thiouridine (s^4^U8), 2-thiouridine derivatives (xm^5^s^2^U34), 2-thiocytidine (s^2^C32), 2-methylthio-*N*^6^-isopentenyladenosine (ms^2^i^6^A37) or 2-methylthio-*N*^6^-threonylcarbamoyladenosine (ms^2^t^6^A37) and 2-thiouridothymidine (s^2^T54) in thermophiles (Figure 2) [9,10]. Thionucleosides are often formed by replacing the keto-oxygen on the base with sulfur [11,12], the exception being for that of thiolated adenines, which are modified with a methylthio-group originating from *S*-adenosyl-l-methionine (SAM) and a sulfur source that is not yet fully agreed upon [13,14,15,16,17]. These sulfur decorations are located throughout the tRNA “L-shaped” tertiary structure (Figure 1) and are important for maintaining proper tRNA conformation and functions. Typically, the functions of given tRNA modifications can be predicted based on their locations within the tertiary structure. Modifications occurring within the anticodon stem loop generally serve a role in translational accuracy and/or efficiency [18,19,20,21], while those located within the acceptor stem, the T-loop and the D-loop often function to maintain structural stability [19,22,23,24], or to serve as a recognition element for the appropriate tRNA aminoacyl synthetase and/or certain modification enzymes [24,25,26,27].

Although thio-modifications are present and retain a variety of functions in all three domains of life, in this review, we focus specifically on their biosynthesis and functions in bacteria, which can vary remarkably between Gram-positive and negative microbes. The occurrence of these modifications as well as the mechanisms governing their biological formation have been enabled through development of methods for detection and quantification of nucleoside modifications in biological samples and in biochemical in vitro reactions. We provide here a comprehensive summary of the available techniques for tRNA isolation and characterization, and highlight the coordination between modification positions within the tRNA structure and their corresponding functions.

### 1.1. Anticodon Stem Loop Modifications

tRNA acts as an adapter molecule between the nucleic acid blueprint and the protein product by bringing amino acids to the ribosome for incorporation into a growing peptide. Thus, it is intuitive that modifications altering the structure and/or reactivity of this biomolecule would have a direct impact on the translational process. Particularly, modifications located in or around the anticodon, such as those at the wobble position 34, as well as positions 32, and 37, generally serve to alter translation, and commonly increase translational accuracy by preventing the occurrence of frameshifting [6,28,29,30,31,32,33,34].

It is generally accepted that 20 universal amino acids are decoded by 61 combinations of triplet codons, in which case, one amino acid could be translated by more than one triplet codon. The first two base pairs in the mRNA codon are usually Watson–Crick paired with nucleotides 36 and 35 in the tRNA anticodon, while the third base can wobble to pair with other nucleotides of mRNA by means of nonstandard interactions [35,36]. Multiple modifications, including methylation and/or thiolation, can occur on position 34 of tRNA to ensure not only specificity, but also flexibility to discriminate multiple codons (Figure 2, Table 1). For instance, the bulky 2-thiocarbonyl group of xm^5^s^2^U flips the 2′-hydroxyl ribose group and fixes the sugar into the C3′-endo structure, locking its interactions to purines and preventing misreading and frameshifting events [9,35]. The thiol group of xm^5^s^2^U also serves as an identity element for certain tRNA synthetases and enhances codon recognition in the ribosome [31,35,37,38,39].

Thiolation of U34 serves a variety of functions in eukaryotes [24,40]. In yeast, wobble uridine thiolation is sensitive to nutrient availability, and when sulfur-containing amino acids are limiting, s^2^U levels are depleted. This downregulates many processes involved in growth and metabolism, the components of which are encoded by genes enriched in codons read by thiolated uridines [41]. Absence of s^2^U showed development defects in eukaryotic model species [42,43]. In humans, mutations within s^2^U biosynthetic genes are associated with acute infantile liver failure and respiratory defects that lead to dysfunction of the auditory system. Furthermore, depletion of s^2^U in humans can lead to mitochondrial encephalopathies, causing epilepsy, ataxia, and dementia [44,45,46,47,48,49]. Interestingly, eukaryotes utilize two distinct pathways for s^2^U biosynthesis in the cytoplasm and the mitochondria, in which only the latter is evolutionarily related to the pathway for this modification in bacteria [24,50,51]. Not surprisingly, bacteria with deficiencies in s^2^U biosynthesis also exhibit severe growth defects [11,52,53,54].

Modifications at position 37 are proposed to tune the stability of codon–anticodon interactions. Recent work has identified a connection between ms^2^t^6^A37 deficiency and type II diabetes due to inefficient and inaccurate translation of several proteins, including proinsulin [55,56]. This altered form of proinsulin is unable to be converted into insulin, leading to glucose intolerance. The impact of A37 modification deficiency on protein synthesis is further recognized by the mitochondrial translational defects resulting from absence of ms^2^i^6^A37 in mice and humans, leading to cardiac dysfunction, accelerated myopathy and respiratory defects [57]. The relevance of tRNA modifications in human disease is intriguing, as they highlight the functional importance of these modifications on a grand scale and provide attractive targets for therapies in disease.

### 1.2. Modifications within tRNA Body and Acceptor Stem

Modifications found within the stem loops of the tRNA commonly affect tRNA structure, folding and stability, while modifications occurring at various positions ensure tRNA recognition by aminoacyl-tRNA synthetases [58,59]. These rules are applicable for most, if not all, tRNA modifications, and hold true for thio-modifications as well. s^4^U, a well-studied photosensor of near-UV light, is a thionucleoside at the intersection of the tRNA acceptor- and D-arms that is highly conserved among bacterial species. When the cell is challenged by near-UV light, s^4^U forms a cross-link bond with cytosine 13, resulting in a shorter distance between acceptor arm and consequently disordering the D-loop and disordered “L”-shape of tRNA tertiary structure [60]. As the structurally changed tRNA becomes a poor substrate for aminoacylation, the cellular translational process is paused and cells enter the stringent response [61,62]. Only present in thermophiles, s^2^T is found at position 54 on the TΨC loop and stabilizes the overall tRNA structure at elevated temperatures [9,40,63].

## 2. Methods for Investigation and Quantification of tRNA Modifications

Analysis of in vivo tRNA modification levels using any of the detection methods described here first requires the isolation and purification of tRNA (or total RNA) from cultured cells and subsequent analysis either using full length tRNA or digested nucleosides. Here, we provide an overview of various methods used for isolation of tRNA from biological samples, and detection of sulfur-containing modifications (Table 2). In many cases, these methods can also be used in the analysis and quantification of other modifications to tRNA which are often used as denominators to determine the relative levels of thio-modifications or in assessing the cellular profile of certain protein expression (see discussion below).

### 2.1. Isolation of tRNA from Biological Samples

There are several methods reported for the isolation of tRNA adjusted to laboratory budget and scale. Thus, while methodologies vary between laboratories, the underlying principles remain constant. First, cells are lysed or homogenized using TRIzol (phenol, chloroform, guanidinium thiocyanate) with agitation, or mechanically, using a bead beater or French pressure cell in presence or absence of lysozyme. Denaturants such as phenol and chloroform, and chaotropic agents, like guanidinium thiocyanate, also quench biochemical processes during isolation, thereby maintaining RNA integrity. Commonly, cells are suspended in aqueous buffer prior to homogenization and subjected to separation between aqueous and organic phases. The use of acidic phenol (pH ~4.5) enables retention of RNA in the aqueous phase, while DNA is extracted into the organic phase. Once RNA is sufficiently isolated into the aqueous phase, it can be further purified using lithium chloride treatment which precipitates large RNA fragments, but not tRNA or small RNA fragments [115]. The resulting soluble material can be precipitated with isopropanol or ethanol, or retrieved with silica-based spin columns. While alcohol precipitation is certainly more cost-effective, yields are lower, and caution must be taken not to over or not sufficiently dry the resulting RNA pellet. Thus, although the RNA isolation process can be flexible, caution is advised to ensure efficient isolation of pure, high quality tRNA samples. This can be evaluated using UV absorbance, denaturing gel electrophoresis, or fluorescent dye-based electrophoresis [116].

Many studies require the purification of individual tRNA species, and advances in liquid chromatography (LC) techniques have streamlined this process as opposed to the cumbersome traditional methods involving gel electrophoresis. There are several chromatography techniques facilitating RNA purification including anion-exchange and ion-pair reverse phase chromatography [116]. Affinity-based approaches for purification of specific tRNA species make use of oligonucleotide probes designed to capture the tRNA isoacceptor of interest using a unique sequence complementary to that within the tRNA [117,118,119]. Though the presence and locations of modifications within tRNA species are not fully known across organisms, the genomic sequences encoding tRNAs are commonly available, and thus can be used for probe design. Affinity purification methods make it possible to isolate bulk amounts of a specific tRNA isoacceptor, which is particularly useful when assessing modification function by comparing structure and/or function of synthetic hypomodified tRNA to biologically isolated and thus, fully modified tRNA species.

In vitro transcription methods to generate synthetic tRNA transcripts enable efficient and robust production of a pure single tRNA isoacceptor. However, the products are completely devoid of modifications, and, in some cases, subsequent kinetic analysis of these transcripts can skew assay results away from physiological relevance. Recent reports have demonstrated that modifications not only modulate the structure and function of these adapter molecules, they also decorate tRNA molecules to mediate specificity in substrate recognition by their enzyme interacting partners. One example is that of a study in which Rodriguez-Hernandez et al. conducted an exhaustive structural and functional investigation of the role of sulfur in tRNA [6]. Aminoacylation and ribosome utilization in *Escherichia coli* was assessed when using synthetic unmodified tRNA transcripts and fully modified tRNA obtained via affinity purification. Structural work showed that hypermodified tRNA^Gln^ induces conformational changes in glutaminyl-tRNA synthetase (GlnRS) which improve the conformation of a surface loop within the protein, and create a specific binding pocket for the 2-thio moiety. Aminoacylation kinetic analysis demonstrated that *E. coli* GlnRS had a 10-fold improvement in binding affinity for tRNA^Gln^ containing s^2^U compared to unmodified tRNA^Gln^, which was further improved five-fold with the addition of the cmnm^5^-modification. Furthermore, thiolated U34 tRNA improved binding affinity to Gln codons and demonstrated five-fold enhancement of GTP hydrolysis by *E. coli* EF-Tu (elongation factor thermo unstable) compared to the unmodified tRNA [6]. Together, these findings provide excellent examples of the effects of tRNA modifications on enzyme kinetics and highlight the importance of comparing results from methods using both synthetic and native tRNA molecules.

#### 2.1.1. Chemical Labeling

Historically, chemical reagents have been used to selectively label modified nucleosides based on the chemical functionality of such modifications [120]. With the rise of mass spectrometry and other sensitive techniques, chemical labeling and modification conversion techniques remain useful as they provide improved detection and chemical stability for target analytes. Thiol groups are strong nucleophiles able to react with a wide range of reagents, including halo-acetamides (s^2^U, s^4^U) [121], 4-bromomethyl-7-methoxy-2-oxo-2*H*-benzopyran (bromomethylcoumarin) [122], and 3-carboxy-2,2,5,5-tetramethyl pyrroline-1-oxyl anhydride with ethyl hydrogen carbonate (mnm^5^s^2^U) [121]. Labeling and detection of other non-thiolated modifications has also been described in the literature. For example, isothiocyanate and activated amines (e.g., ethylenediamine) can covalently interact with the carboxyl group on t^6^A [123] and aliphatic amines can attack queuosine [124]. Although these reagents have specific selectivity towards target modifications, other modifications sharing similar functional groups can display cross-reactivity. For instance, in addition to bromomethylcoumarin’s ability to target thiolated uridine [122], it also reacts with pseudouridine [125], and potentially with uridine and thymidine [126].

#### 2.1.2. Enrichment of Modified Nucleoside

Thiophilic “soft” metal mercury reacts readily with sulfur-modified nucleic acids. Igloi and his colleagues in 1988 first introduced a synthetic [(*N*-acryloylamino)phenyl]mercuric chloride (APM), which can be co-polymerized into polyacrylamide gels to separate thiolated tRNA [127]. APM was further developed by the Biondi group into a three-layer polyacrylamide gel with only the middle layer containing a high amount of the organomercuric compound [128]. The formation of a coordinate covalent bond between Hg and S ligand retards the electrophoretic migration of thio-modified RNA. The results can be visualized by standard methods for polyacrylamide electrophoresis imaging, including fluorescence, silver or ethidium bromide staining. Although the APM-gel has proved to be a low-cost and effective detection method, different thiol-containing tRNAs have shown different migration patterns through the APM layer due to varying structures of the thionucleotide. The APM-gel up to date has shown a great ability to covalently link 4-thiouridine and 2-thiouridine and its derivatives. However, when the sulfur is not in the thiocarbonyl form, for instance, in ms^2^i^6^A or ms^2^t^6^A modifications, APM has not been found to covalently coordinate with these thio-modified nucleosides effectively [129]. Despite its low selectively towards different sulfur modifications in tRNA, APM gel separation is still one of the most commonly used detection and isolation methods for thiolated RNA.

Queuosine is a non-thiolated modification, but its biosynthesis depends on [Fe-S] clusters (Table 1). This modification can also be retained in a polyacrylamide gel containing a boronic acid derivative. The synthetic *N*-acryloyl-3-aminophenylboronic acid (APB), when co-polymerized into acrylamide gel, is able to retain *cis*-diol groups present in tRNA, like queuosine, resulting in separation of the *cis*-diol containing ribonucleic acid [130,131]. However, APB is not only able to separate queuosine, but any tRNA samples containing *cis*-diol, including RNA with non-phosphorylated 3′ ends [130] and the newly discovered bacterial nicotinamide adenine dinucleotide (NAD)-capped tRNA [132]. From both APM- and APB-gel detection, thionucleosides may be extracted and isolated for further quantification.

#### 2.1.3. Northern Blot

Northern blot has been a valuable tool for probing individual tRNA species. Sequence specific hybridizing probes are usually modified with either radioisotope ^32^P or fluorescent labels to improve detection sensitivity. Although radioactive-labeled probes are advantageous for improving detection sensitivity and assay quality, the disadvantage of such experiments is the need to minimize the usage of radioactive substances. This technique has also been used in conjunction with APM-containing polyacrylamide gels to identify the occurrence of thiolation on certain tRNA species [133,134,135,136,137]. Recently, a variation of the standard method, immuno-Northern blot, was provided which uses antibodies that specifically bind with modified nucleosides such as 1-methyladenosine (m^1^a), *N*^6^-methyladenosine (m^6^A), pseudouridine, and 5-methylcytidine (m^5^C) [138]. This highly sensitive and relatively simple protocol enables small laboratories to compare the abundance of modified nucleic acids across samples.

### 2.2. Quantification of tRNA Modification Levels Using Liquid Chromatography

Detection and quantification of tRNA modification levels using liquid chromatography (LC) is performed on nucleosides isolated from purified tRNA samples. In this case, bulk tRNA needs to be first digested into individual nucleotides using nuclease P1 or endonuclease Bal31 followed by treatment with phosphatase [116,139,140]. Individual nucleosides are then subjected to separation via reverse phase high pressure liquid chromatography (RP-HPLC). Commonly used protocols for digestion of tRNA involve sequential incubation with acidic and basic buffers for nuclease P1 and alkaline phosphatase, respectively. However, recent studies have reported that both acidic and basic reaction conditions lead to degradation of many labile modifications, such as ct^6^A to t^6^A and ms^2^ct^6^A to ms^2^t^6^A [59,87,88]. Alternatively, this problem can be overcome by digestion at physiological pH, and the use of endonucleases like benzonase, which is active at physiological pH [116].

#### 2.2.1. High Pressure Liquid Chromatography Separation Coupled to Ultraviolet-Visible Detection

The absorption maxima of most nucleosides lies around 260 nm. However certain modifications to the nitrogenous bases alter the physicochemical properties of specific nucleosides, resulting in distinct maximum absorption wavelength (λ_max_) values, including for many thionucleosides (s^2^U λ_max_ ~280 nm, s^4^U λ_max_ ~330 nm, s^2^C λ_max_ ~245 nm, ms^2^A λ_max_ ~240 nm) [139,140]. Therefore, RP-HPLC coupled to UV-Visible detection, particularly with a diode array detector, has been extremely useful in past decades for quantification of such modifications. For this analysis, C18 columns are used to separate nucleosides starting with a high percentage (98%–100%) of aqueous solvent to elute the most polar nucleosides, followed by a gradient of organic solvent, such as acetonitrile or methanol to elute those with more hydrophobic properties. Considering that nucleosides are intrinsically polar, general C18 columns are not always sufficient for separation of certain nucleosides, such as pyrimidines and their derivatives. While addition of 10 mM sodium or ammonium acetate into the aqueous buffer at a pH ~4.5 enhances the separation and resolution of individual nucleosides, the chromatography parameters must be tailored to the individual needs of each study, focusing on complete resolution of the analytes of interest. This concern has spurred the application of hydrophilic interaction liquid chromatography (HILIC), in order to increase retention and resolution of nucleosides carrying polar modifications [91,141,142]. Though LC with UV-Visible detection has been an invaluable tool for analysis of tRNA modifications in years past, it is limited by sensitivity and the requirement of commercially available standards (Table 3) to validate retention times. The recent discovery of several previously unidentified nucleoside modifications has exacerbated this challenge, as manufacturers have not yet caught up to the field in terms of providing a complete set of hyper-modified nucleosides on the market, including many nucleosides which are dually modified.

#### 2.2.2. Advances in Liquid Chromatography–Mass Spectrometry Methods for Detection of tRNA Modifications

Analysis of modified tRNA nucleosides using LC techniques coupled to mass spectrometry (LC–MS) has become increasingly popular. The recent discovery of novel modifications previously overlooked by methods described above is attributed to the recent developments in both instrumentation and technology for data analysis. In addition to detecting and quantifying an ion of a precise molecular weight with great sensitivity, fragmentation of the ion of interest, using tandem LC–MS/MS simultaneous enables structural validation and identification of multiple analytes (Table 3). For relative quantification, modified nucleosides occurring in great abundance such as pseudouridine, dihydrouridine, and inosine are often used as internal standards to normalize modification levels between samples. However, it is important to take into consideration the tRNA isolation method and degree of purification, as some of these modifications occur in additional RNA species and thus small degrees of contaminants could widely skew results. Of the tRNA nucleosides listed in Table 3, pseudouridine, dihydrouridine, m^2^A and m^6^A are all found in bacterial rRNA [143,144,145,146,147,148]. This leaves inosine as an attractive internal standard candidate. Although it has only been observed in bacterial tRNA, it is important to note that both inosine and m^6^A also occur in eukaryotic mRNA [149,150].

Typically, identification of new modified nucleosides is enabled with the use of tandem triple quadrupole (QQQ) or high mass accuracy mass spectrometers (LTQ-Orbitrap) with an electrospray ionization (ESI) source in positive-ion mode, as both aqueous (water) and organic (acetonitrile or methanol) solvents have 0.1% formic acid added to facilitate ionization. Tandem MS/MS is used to fragment the ion, and neutral loss analysis provides a way to identify bases originating from nucleosides, as they lose an uncharged mass associated with a ribose ring (Table 3). Although quadruple time of flight (Q-TOF) and Orbitrap high mass accuracy mass spectrometers are sufficient for relative quantification, the use of a tandem QQQ LC-MS/MS with ESI is advised for absolute nucleoside quantification, as its capacity for sensitivity is superior to that of other instrumentation. Supplementation of an additional and complementary Q-TOF MS/MS method and/or various nuclear magnetic resonance (NMR) spectroscopy techniques can also aid in structural assignment [116,152,153].

LC–MS/MS has also demonstrated its utility in tRNA analysis through methods that determine the exact position at which a modified nucleoside occurs within one or more tRNA species. One strategy is to analyze tRNA isolated through affinity purification and subsequently digested into nucleosides. Alternatively, total tRNA can be digested with one or more sequence specific RNase(s) to yield oligonucleotides of predicted sizes. These oligonucleotides can then be applied to a C18 column coupled to a QQQ MS with ESI in negative mode for LC-MS/MS detection. Online resources, such as the Mongo Oligo Mass Calculator, facilitate easy predictions of expected masses based on instrument ionization settings and RNase(s) used for digestion [119,154,155].

In 2010, Chan et al. made use of the aforementioned LC-MS techniques to develop a systems-level approach for studying dynamic changes in tRNA modification levels in *Saccharomyces cerevisiae* cells exposed to various stresses [156]. This and subsequent studies resulted in the discovery that in stress conditions, altered tRNA modification levels directly impact synthesis of codon-biased stress response proteins [118,152,157,158]. For this technique, tRNA isolated from cells exposed to the stress condition of interest was isolated, purified, digested into nucleosides and quantified with LC–MS/MS. Upon data normalization and multivariate statistical analysis, a map of significant changes within tRNA modifications was generated. Recent reports have provided a comprehensive review of the methods involved in the systems-level approach for tRNA modification analysis [116,159]. Altogether, emerging advances in LC-MS techniques for RNA modification analysis have revealed underappreciated roles for tRNA modifications in cellular metabolism. These findings suggest that we have only begun to scratch the surface of their biological relevance, thus providing a direction for future research and simultaneously demonstrating the value of investing in tools to facilitate analysis of biological samples.

## 3. Biosynthesis of Thionucleosides in Bacterial tRNA

Methods for detection and quantification of tRNA from biological samples such as the ones described in this review have been critical tools for the analysis of thio-modifications. Investigation of pathways involved in the synthesis of thio-modifications of tRNA as well as discovery of new intermediates in these pathways were afforded through a combination of genomics, genetic, and biochemical approaches validated with spectroscopic and analytical methods for structural identification and quantification of these reaction products. Here, we summarize the current knowledge on the biological synthesis of major thionucleosides in bacteria.

### 3.1. 4-thiouridine (s^4^U)

Sulfur-modification on the uridine at position 8 of tRNA has been known for over half a century [160]. It serves as a photo-sensor in bacteria and archaea for cells mediating the stringent response under environmental UV stress. Due to its unique λ_max_ at 330 nm, s^4^U has also been explored as a photoreactive ribonucleoside analog in RNA synthetic studies [161]. There are currently three synthetic pathways for the biosynthesis of s^4^U in Bacteria and Archaea. The first pathway reported is the one utilized by the model Gram-negative bacterium, *E. coli*, and it has provided extensive mechanistic details involving nucleoside thiolation and general persulfide transfer reactions. The synthesis of this modification involves the sulfur activating enzyme IscS and the dually functional thiouridylase ThiI (Table 1) [11,12,62,64,66,162]. Earlier studies in *Salmonella enterica* have shown that both enzymes also participate in the synthesis of thiamine. In both pathways, IscS uses pyridoxal 5′-phosphate (PLP) to activate the amino acid cysteine, enabling the formation of an enzyme persulfide intermediate, and the release of alanine. The subsequent step in this reaction mechanism is the transfer of persulfide sulfur to an acceptor molecule. In the biosynthesis of s^4^U and thiamine, the Cys456 residue of ThiI, residing within the rhodanese domain, serves as the sulfur acceptor site [70]. Interestingly, in thiamine formation, this C-terminal rhodanese domain is both necessary and sufficient for the function of ThiI in this pathway [66].

**Figure 3:**
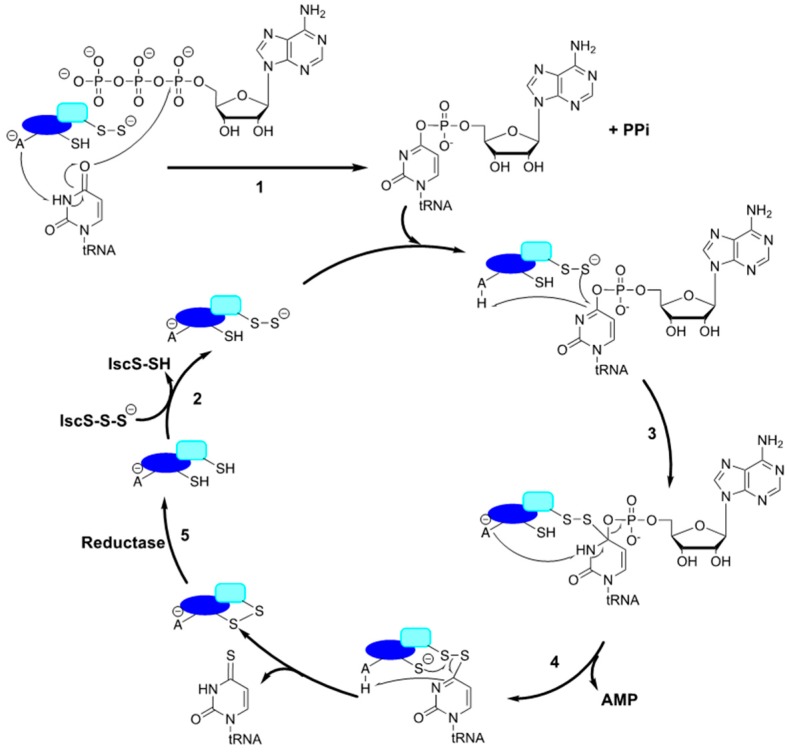
Proposed mechanism for thiolation of tRNA U8 by *E. coli* IscS and ThiI (adapted from [64,162]). The ThiI thiouridylase domain (dark blue) catalyzes the ATP-dependent adenylation of C4 of uridine and release of pyrophosphate (PPi) (1). IscS promotes persulfide sulfur transfer to Cys456 of ThiI’s rhodanese-like domain (light blue) (2). In one of the proposed mechanisms, the persulfide sulfur then conducts a nucleophilic attack onto the activated C4 of U8 tRNA (3), which leads to release of the leaving group AMP (4). The reaction is then resolved through formation of a disulfide bond between Cys456 and Cys344 and release of the product s^4^U tRNA (5). Regeneration of the enzyme for subsequent catalytic cycles requires the involvement of a yet unidentified reductase (5). An alternative proposal for the formation of s^4^U involves the local formation of sulfide at the enzyme active site which then directly attacks the adenylated intermediate (not shown). In both models, the reaction cycle is marked by the release of AMP and the s^4^U tRNA product along with the formation of a disulfide bond between Cys344 and Cys456.

Specific to s^4^U synthesis, the activity of ThiI also depends on other structural and functional domains located towards the N-terminus. While the C-terminal rhodanese sulfur transfer domain appears to be missing in certain ThiI sequences, structural and sequence analyses reveal the presence of three additional majorly conserved domains [67,69] comprehending a large thiouridylase domain. The THUMP (named after thiouridine synthases, RNA methylases and pseudouridine synthases) domain is involved in recognition of the 3′-CCA end of tRNA and positioning the uridine in the correct orientation for catalysis [67]. The N-terminal Ferredoxin-like domain (NFLD) is also involved in the binding of tRNA [69], while the C-terminal PPase domain contains an adenylation-specific PP-loop motif (SGGFDS) belonging to the PP_i_ synthetase superfamily [65]. This signature motif is also found in other tRNA adenylating enzymes, including the 2-thiocytidine and 2-thiouridine biosynthetic enzymes, TtcA and MnmA, and uses ATP to activate the appropriate uridine carbonyl groups in substrates [94,163]. Thus, the adenylation step serves to introduce a good leaving group for the following nucleophilic attack of sulfur intermediate species (Figure 3).

The proposed mechanism first evokes the ThiI adenylation reaction of the C4 of tRNA U8, followed by the sulfur transfer reaction by the persulfurated form of ThiI with the concomitant release of AMP. Two reaction paths have been proposed for the final sulfur transfer step [162]. The mechanism described in Figure 3 involves the direct nucleophilic attack of the persulfide sulfur onto the adenylated intermediate, which is then resolved by another residue Cys344, thereby regenerating the enzyme for subsequent turnover. The mechanism of s^4^U formation can also be explained by an alternative proposal in which Cys344 acts as a nucleophile by attacking the bridging sulfur of Cys456 leading to local release of sulfide that can directly react with the tRNA intermediate [62,162]. In both schemes, the completion of the catalytic cycle is marked by formation of a disulfide bond between the Cys344 and Cys456. While these reactions can complete one turnover under non-reducing conditions, reduction of the disulfide bond between the catalytic and resolving cysteine residues is necessary for the next cycle. Multiple turnover reactions can only be achieved in vitro in the presence of reductants such as dithiothreitol (DTT), however, the identity of the physiological reductant enabling regeneration of ThiI at the end of each turnover remains to be identified. Additionally, ThiI conserved residues Asp189 and Lys321 were found to be critical for ThiI’s enzymatic adenylation activity and consequently overall function of ThiI in s^4^U formation [163]. Although the aspartic acid residue lying within the PP-loop in PP_i_ synthetases has long been established as for Mg^+^ coordination for ATP binding and hydrolysis [164], the exact roles of the Asp189 and Lys321 residues of ThiI in ATP binding and hydrolysis or the initial deprotonation of *N*5 of U8 (Step 1, Figure 3), need to be further validated.

The pathway for s^4^U in Gram-positive bacteria is distinct from the one described in *E. coli* and *S. enterica*. In fact, the majority of Gram-positive bacteria don’t contain IscS and encode a truncated ThiI protein lacking the rhodanese domain. The *Bacillus subtilis* ThiI appears to be a dedicated protein in s^4^U biosynthesis and it is not required for thiamine generation [165]. Interestingly, *B. subtilis* contains four cysteine desulfurases and evidence thus far has demonstrated that each pairs with a dedicated sulfur transferase for specific thiocofactor generation [68,72,166]. Formation of s^4^U, in particular, is accomplished by the cysteine desulfurase NifZ, the coding sequence of which is found immediately upstream of *thiI*. Though the truncated ThiI protein lacks the rhodanese (Rhd) domain, it contains the NFLD, THUMP and PPase domains involved in adenylation and thiolation of U8 [68]. Despite the missing rhodanese domain, *B. subtilis* ThiI is capable of receiving the persulfide sulfur from NifZ, and both proteins together are sufficient in completing s^4^U synthesis. The *B. subtilis* ThiI contains four cysteine residues, including Cys344 at a position equivalent to *E. coli* ThiI Cys344. However, the involvement of *B. subtilis* ThiI Cys344 and/or an alternate Cys residue serving as the sulfur acceptor site awaits experimental investigation. Nevertheless, based on the proposed mechanism of *E. coli* ThiI involving two cysteine residues, it is possible that more than one Cys residue within *B. subtilis* ThiI uridylase domain is involved in thiolation. Alternatively, due to the presence of a dedicated cysteine desulfurase, it is also reasonable to consider that in addition to promote persulfide transfer, NifZ may also involved in completing ThiI’s catalytic cycle by providing a resolving cysteine at the end of the catalytic cycle.

A third pathway for s^4^U8 tRNA formation was recently reported for the archaeal species *Methanococcus maripaludis* [167]. Like Gram-positive bacteria, most archaeal species do not contain a master/housekeeping IscS-like cysteine desulfurase and ThiI sequences lack the Rhd domain. Mutagenesis studies showed that the three conserved cysteine residues are essential for the function of ThiI, and Ala substitution of any of these residues eliminates the ability of this protein to serve as a sulfur acceptor [168]. Recent spectroscopic studies demonstrated that *M. maripaludis* ThiI is capable of coordinating a [3Fe-4S] cluster which has been assigned as an important element for the functionality of this protein, however the exact role of this cluster as structural, catalytic or a sacrificial sulfur source has not been determined [167].

### 3.2. 2-thiouridine (s^2^U)

The 2-thiouridine modification is located on the third (wobble) position of the anticodon in tRNA^Glu, Gln, Lys^ in all organisms. The widespread conservation of this modification, in addition to the metabolic defects associated with its absence in various species demonstrates the utility of this modification in metabolism. Eukaryotic organisms such as *Saccharomyces cerevisiae* contain two distinct machineries for s^2^U biosynthesis in the cytoplasm vs. the mitochondria. Notable differences in the machineries are the usage of Fe-S proteins and sulfur transfer via protein-thiocarboxylate formation in the cytoplasmic pathway [135,167,169,170]. The mitochondrial pathway, however, resembles that used in bacteria, as it is [Fe-S] cluster independent, and utilizes persulfide sulfur transfer rather than thiocarboxylation [171,172].

#### 3.2.1. *E. coli* 2-thiouridine Biosynthesis: Assembly Line of Sulfur Transfer

The biosynthesis of s^2^U has been extensively studied in *E. coli*, which involves participation of six different enzymes. In both *E. coli* and *S. enterica*, the cysteine desulfurase IscS also serves as the sulfur source by transferring its persulfide sulfur to TusA [37,52]. The small sulfurtransferase TusA then interacts with and transfers sulfur to TusD, when in a heterohexameric (α_2_β_2_γ_2_) complex with additional components TusB and TusC. TusE serves as an intermediate between the TusBCD complex and the thiouridylase, MnmA [37,70]. The latter has dual functions analogous to those aforementioned for ThiI: adenylation and thiolation. TusE can form a ternary complex with MnmA and tRNA and it was initially unclear whether MnmA could thiolate the tRNA following adenylation, or if TusE being in the ternary complex was necessary for sulfur insertion. However, structural and functional studies have confirmed that persulfurated MnmA is both capable and sufficient for adenylation and thiolation of U34 tRNA [71,173].

In Gram-positive bacteria, the biosynthesis of s^2^U utilizes an abridged pathway. Genomic analysis of several Gram-positive species pointed to the absence of sequences encoding Tus proteins. Interestingly, in *B. subtilis* and in several other Gram-positive species missing *tus* genes, an IscS-like cysteine desulfurase gene (*yrvO*) is located in the same genomic region, and commonly directly upstream, of *mnmA*. Experimental characterization of *B. subtilis* YrvO and MnmA both in vivo and in vitro demonstrated that these two proteins are the sole requirements for s^2^U formation in this bacterium [72]. It is worth noting that the *B. subtilis* genome encodes a rhodanese protein (YrkF) with homology to TusA which is a competent sulfur acceptor of YrvO, but inactivation of *yrkF* leads to no changes in s^2^U levels [72,174]. These findings highlight an important difference in thiocofactor biosynthesis between Gram-positive and negative bacteria, in that the presence of a devoted cysteine desulfurase in *B. subtilis* eliminates the need of the intricate Tus sulfur transfer system through direct interaction with MnmA. The conservation of the *yrvO–mnmA* gene region and lack thereof the Tus system suggests that the abbreviated s^2^U biosynthetic pathway used in *B. subtilis* is common to Gram-positive bacteria. In Gram-negative *E. coli*, which uses the housekeeping IscS for sulfur mobilization to all thiocofactors [11,53,54], the Tus proteins also participate in additional metabolic pathways [71,175,176,177]. The multi-functionality of these and other enzymes in thiocofactor pathways has been attributed to the utilization of one major cysteine desulfurase (i.e., IscS) for all thiocofactors in Gram-negative bacteria and simultaneously demonstrates the resulting complexity of sulfur trafficking in these organisms [178,179]. The recruitment of small sulfur acceptor proteins or sulfurtransferase domains within individual pathways can be seen as a mechanistic strategy for controlling sulfur flux in organisms such as *E. coli* that utilize a master sulfur donor. While in other systems, such as the Gram-positive *B. subtilis*, the biosynthetic apparatus is much simpler.

#### 3.2.2. tRNA Binding and Adenylation by MnmA

**Figure 4:**
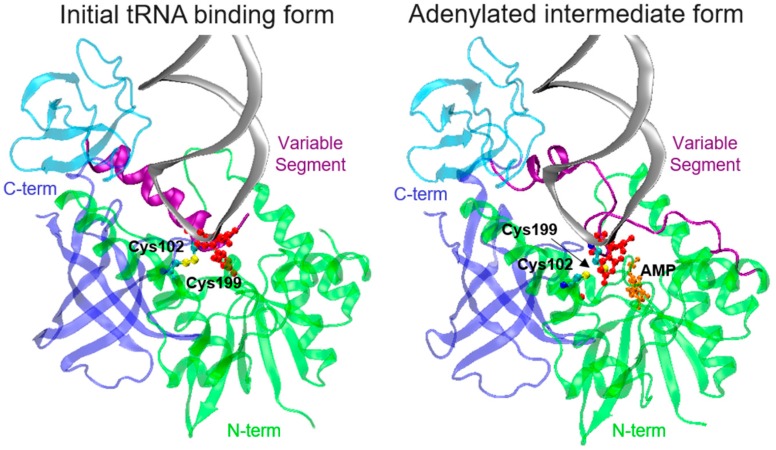
*E. coli* MnmA in complex with tRNA^Glu^. The structure of the *E. coli* 2-thiouridine biosynthetic enzyme MnmA is shown in complex with tRNA^Glu^ (gray) in the initial tRNA binding form (2DER, left) and the tRNA adenylated intermediate form (2DEU, right). *E. coli* MnmA is colored by domain; the N-terminal catalytic domain (residues 4–215) is shown in green, while the central domain (residues 216–277), variable segment (residues 187–215) and the C-terminal domain (residues 278–368) are shown in cyan, purple and blue, respectively. U34 is shown in red and AMP is displayed in in orange. The sulfur atoms of the MnmA catalytic Cys residues, 102 and 199, are shown in yellow. C-term: C-terminal and N-term: N-terminal.

Similar to the ThiI reaction (Figure 3), the thiouridylase MnmA has two functions in addition to acting as a sulfur acceptor protein: adenylation of the tRNA substrate, followed by thiolation at the C2 position of U34. The exact sequence of events for substrate binding and activity is still unclear, however Numata et al. provided detailed insight into this process via three structures of *E. coli* MnmA in complex with tRNA^Glu^ which illustrates the initial binding, the pre-reaction, and the adenylated intermediate states [173]. The structures of the MnmA-tRNA complex showed that in its initial tRNA binding state, MnmA displays an open conformation, with the variable segment coiled back in an α helix, whereas in the pre-reaction and adenylated intermediate states, it undergoes a subtle rearrangement, in which the variable segment is extended, adopting a “closed conformation” to shield U34 from the solvent and position it closer to the catalytic residues (Figure 4) [173]. In both *E. coli* and *B. subtilis*, sulfur transfer to MnmA was only observed in the presence of ATP, suggesting that ATP-bound MnmA is the catalytically competent form of the enzyme [72,173]. The mechanisms by which ATP binding elicits activation of MnmA as a sulfur acceptor are not known. However, it seems mechanistically advantageous for the enzyme to require ATP prior to accepting a persulfide sulfur, to ensure the availability of all necessary reaction components prior to binding tRNA and closing its active site. It is tempting to speculate that ATP binding induces a conformational change in the enzyme to render its active state. However, the only crystal structure in which ATP was present was that of the adenylated intermediate state, meaning that the closed conformation in the *E. coli* MnmA pre-reaction state was not induced by ATP. Interestingly, the ATP dependence for sulfurtransferase activity is not observed in ThiI from either organism [68,162,180]. Considering that *E. coli* ThiI contains a rhodanese sulfurtransferase domain, and that ATP is not necessary for its’ additional function in thiamine biosynthesis, it seems logical that its sulfurtransferase activity is independent of ATP binding [68]. Alternatively, *B. subtilis* ThiI lacks the rhodanese domain and does not participate in thiamine formation, yet its competency in the sulfur transfer event is independent of ATP. Collectively, this suggests that the requirement for ATP binding to MnmA for sulfur transfer is a unique feature of this enzyme, rather than a characteristic common to all thiouridylases.

#### 3.2.3. Proposed Order for tRNA U34 Modification

The U34 modifications at the C2 and C5 positions have been suggested to occur independent of one another. Indeed, neither modification is strictly required to be present in order for the other to be synthesized, as strains deficient in the genes coding for the C5 modifying enzymes accumulate s^2^U and vice versa [181,182]. However, investigation of *B. subtilis* MnmA’s substrate preferences via in vitro s^2^U reconstitution experiments using native tRNA from an *E. coli ΔmnmA* strain, and enriched for mnm^5^U34 tRNA, demonstrated a clear preference for modification of hypomodified U34 [72]. Evaluation of the MnmA–tRNA complex structure in the pre-reaction state (i.e., closed conformation; Figure 4, right) shows that the Phe154 and Asp99 are positioned 4 Å away from C5 of the U34 and that C5 provides a van der Waals interaction with the Phe154 aromatic ring. Furthermore, MnmA’s tRNA substrates also contain A37 modifications, and when t^6^A37 was superimposed on the structure, it was proposed that it might provide contacts to stabilize the MnmA-tRNA interaction, suggesting that A37 modification(s) may precede U34 thiolation [173]. Altogether, the substrate preference of *B. subtilis* MnmA for hypomodified U34 tRNA, along with the steric hindrance potentially imposed by the mnm^5^ group, which would be compounded in an A37 modified tRNA anticodon suggest that a sequential order for U34 modification exists, with thiolation preceding C5 modification. In the past decade, significant progress has been made towards understanding the intricacies of s^2^U biosynthesis. However, differences in experimental design of studied systems often explored through in vitro reactions using hypomodified synthetic transcripts make it difficult to draw general conclusions about the order of modifications. Therefore, future work must capitalize on the powerful analytical tool provided by evaluation of both synthetic and biological tRNA samples using newly developed sensitive and accurate techniques for detection of modifications and associated intermediates present at low abundance.

The s^2^U34 modification at the C2 position can be further modified either by addition of a geranyl group, or the sulfur can be substituted for selenium, but both modifications require thiolation of U34 as a precursor. Interestingly, in *E. coli* both modification reactions are catalyzed by the MnmH (SelU/SufY/YbbB) rhodanese domain containing protein [81,183]. It is not surprising that a rhodanese protein can substitute sulfur for selenium, as both atoms are similar in structure and physicochemical properties. Yet, the geranyl modification is entirely different (Figure 2), as it is a large hydrophobic moiety and could influence the structure and function of the tRNA, thereby impacting translation [83,184]. In fact, the geranyl group lends specificity by preferentially binding codons ending in G [29]. It is estimated that approximately 3%–7% of s^2^U34 tRNAs are geranylated at a given time, and this can vary, as selenation rather than geranylation is observed at selenium concentrations above 10 nM. It was initially proposed that geranylation is an alternative to selenation when Se is limiting [29]. However, co-purification of MnmH with an enriched geranyl-modified tRNA population bound to it [80,81] suggests that geranylated tRNA may in fact serve as an intermediate for selenation in vivo. This could also explain the significantly low levels of ges^2^U under normal growth conditions. Although in vitro selenation reactions have demonstrated that geranylation is not required for synthesis of se^2^U [80], it seems likely that the sulfur atom in its thioether form with a bulky geranyl group attached would be a better leaving group than sulfur in its thioketone form. Thus, perhaps geranylation of s^2^U enhances the turnover rate of MnmH’s selenation activity. Kinetic characterization of MnmH’s dual activities and substrate preferences alone or in competition assays would certainly enable clarification of the sequence of reactions performed by this enzyme. The physiological relevance of the se^2^U modification stems from the narrow range in which Se is beneficial (0.1–1 µM). Similar to sulfide, free selenide is highly reactive, and levels above 10 µM are toxic to many bacteria, providing a rationale for detoxification mechanisms [185]. Although it is well known that Se incorporation into certain enzymes in the form of selenocysteine (Sec) is important for high enzymatic activity, the function of se^2^U is poorly understood. Considering the similar structural and physicochemical properties of sulfur and selenium, it seems redundant for the cell to convert one modification to the other if their functions are the same. An alternative explanation for se^2^U formation would be as a way of shuttling Se into a non-toxic form, by converting Sec pools to se^2^U, thus having a smaller impact on intracellular redox and preventing its interference in normal sulfur metabolism, while maintaining the initial function of the C2 modification of tRNA U34.

Recent studies investigating the mechanism of catalysis for MnmH’s two distinct activities have shed light on the contributions from this protein which dictate formation of each modification. Though the conserved Cys97 in the rhodanese domain is required for selenation of U34, this same residue is not necessary for geranylation [80]. There is however, a 36 amino acid insertion sequence in the rhodanese domain of MnmH orthologues that when altered, increases geranylation. This insertion is not found in single domain rhodanese proteins and analysis of many different MnmH variants altered within this region suggested that the size of residues, rather than its chemical properties, were important for enhanced geranylation [81,152]. Another interpretation is that mutation of residues within the rhodanese domain prohibit further modification from ges^2^U to selenation, and consequently the higher extent of geranylation represents intermediate build-up. An intact Walker motif in the MnmH P-loop domain is proposed to be the binding site for the substrate geranylpyrophosphate (GePP), while the domain itself is necessary for tRNA binding and thus for both activities. Altogether, the two distinct activities of MnmH are apparent and involve either distinct or potentially sequential chemistries, and further exploration will be required to deconvolute this biosynthetic pathway and place it in a cellular context. In addition to *E. coli*, other species containing MnmH and consequently carrying geranyl s^2^U include *Enterobacter aerogenes*, *Psdeudomonas aeruginosa*, and *Salmonella typhimurium* (Table 1). Not surprisingly, this modification was not detected in *Vibrio fischeri*, *B. subtilis*, or several different eukaryotic organisms lacking the *mnmH* gene [81].

### 3.3. 2-thiocytosine (s^2^C)

Thiolation of cytosine at the C2 position (s^2^C) is a thio-modification that is not as well studied as other modifications described in this review. This thionucleoside has been reported in bacteria and some archaeal species, including *Thermococcus* sp. [186,187]. This modification is present at position 32 of only four tRNA species (tRNA_ICG_, tRNA_CCG_, tRNA_mnm5UCU_, and tRNA_GCU_) in *E. coli* and *S. enterica* [63]. Like other thionucleosides, sulfur from cysteine is activated through a cysteine desulfurase as the ultimate sulfur source for s^2^C formation. Initial work showed that the formation of s^2^C along with ms^2^i^6^A/ms^2^t^6^A not only requires IscS but also depends on the activity of the [Fe-S] cluster scaffold protein IscU, indicating the role of [Fe-S] clusters in these biosynthetic pathways. Besides the requirement of [Fe-S] cluster biosynthetic genes, the biosynthesis of s^2^C also requires the product of the *ttcA* gene [63]. The TtcA sequence bears a ^39^SGGGKDS^45^ PP-loop, the structural motif belonging to the ATPase superfamily which also includes the aforementioned ThiI and MnmA thiouridylases. Therefore, it was proposed that TtcA also uses an adenylation mechanism similar to that of ThiI, MnmA, TtuA, and TilS to activate the carbonyl substrate and yield a tRNA–AMP intermediate (Figure 3).

The events leading to subsequent sulfur insertion into the C2 position of tRNA C32 are less understood. Isolation of TtcA showed the presence of a [4Fe-4S] cluster likely coordinated by three Cys residues. Mutagenesis studies showed that Cys 122, 125, and 222 are critical for cluster accumulation and in vivo function of TtcA. Similar to the archaeal [Fe-S] cluster-containing thiouridylase ThiI, the involvement of the cluster as either the source of sulfur in a sacrificial role, or serving as an intermediate ligand for sulfur, or perhaps simply as a structural element is not clearly defined [63,94]. Notably, ATP and a reducing agent like DTT are absolutely required for in vitro s^2^C reconstitution.

### 3.4. Modifications within Adenosine 37

The 37 position of tRNA, neighboring the anticodon, is always a purine and throughout all three domains of life, it is commonly modified [3,50]. Several forms of this modification have been discovered (Figure 2), that serve multiple purposes and all seem to stabilize and secure the first base pair interaction between codon and anticodon of A:U/U:A, thereby preventing frameshifting [24,39]. Furthermore, A37 modifications help to structure the anticodon loop in an open conformation for proper decoding by preventing intraloop base pairing [188]. Methylthiolation at C2 of A37 always occurs in conjunction with a modification at the *N*^6^ position with either an isopentenyl-(i^6^) or threonylcarbamoyl-(t^6^) group. The *N*^6^-isopentenyladenosine (i^6^A) modification is found in all domains of life, and in bacteria it can be further modified to include a methylthio group at the C2 position, yielding ms^2^i^6^A and in some species, the hydroxylated derivative ms^2^io^6^A. The bacterial enzyme MiaA is responsible for transfer of the dimethylallyl moiety from dimethylallyl pyrophosphate to C6 of A37 following recognition of its A_36_A_37_A_38_ sequence containing tRNA substrate [189,190].

The A37 position in tRNAs which decode ANN codons is universally modified to t^6^A [191]. Flies, plants, mammals, and some γ-proteobacteria can further methylate the *N6* position of A37 in a SAM-dependent reaction by the tRNA methyltransferase TrmO to produce m^6^t^6^A [50,88,192]. The threonyl modification is important for translational accuracy and codon recognition as its hydrophobic moiety forms a large planar structure which facilitates base stacking and strengthens binding of A:U base pairs as mentioned above [39,191]. Additionally, the role of this modification in AUG start codon selection provides a justification of the reason for evolutionary conservation of this modification across all organisms. Several genes are necessary for biosynthesis of this modification; in *E. coli*, the *tsaC, tsaD, tsaB,* and *tsaE* are associated with formation of t^6^A [11,193,194,195].

In some groups of bacteria, such as *E. coli* and *B. subtilis*, as well as fungi, plants and protists, a cyclic form of t^6^A (ct^6^A) has been observed, which also enhances tRNA decoding activity. Recently, X-ray crystallography, along with LC–MS analysis of native and synthetic ct^6^A enabled further characterization of the nucleoside in the hydantoin isoform, rather than the previously predicted oxazolone isoform (Figure 2), with a λ_max_ of 269 nm. From the structure, it is not intuitive how the twisted position of the ring against the adenine base contributes to decoding efficiency and less understood are the effects of this further modification on translation [87,88].

In *E. coli*, formation of ct^6^A from t^6^A is catalyzed by threonylcarbamoyladenosine dehydratase A (TcdA), previously known as CsdL (*B. subtilis* YrvM). TcdA catalyzes an ATP-dependent dehydration reaction of t^6^A [59]. The proposed reaction mechanism likely involves the activation of the carboxyl group of the t^6^A threonyl moiety through adenylation. This step then enables the nucleophilic attack of the *N6* nitrogen of t^6^A onto the activated carboxylate group, leading to formation of the hydantoin ring [87]. Interestingly, in *E. coli* the *tcdA* gene is located adjacent, yet in the opposite direction of *csdA* and *csdE* genes encoding a cysteine desulfurase and sulfur acceptor, respectively. Although this modification does not appear to involve a sulfur transfer reaction, deletion of either gene severely impairs the levels of ct^6^A and thus, the involvement of the cysteine desulfurase CsdA and sulfur acceptor CsdE in ct^6^A formation remains not understood [59,85]. Interestingly, *B. subtilis*, *Trypanosoma brucei* and three plant species were recently found to contain ms^2^ct^6^A, which was synthesized by their TcdA and MtaB enzymes. Results from investigation into ms^2^ct^6^A synthesis demonstrated that cyclization of t^6^A can occur either at t^6^A or ms^2^t^6^A, and suggested that the levels of this hypermodification can be regulated according to specific physiological conditions. Nevertheless, while no phenotypes have been identified thus far for inactivation of either *tcdA* or *mtaB* in *B. subtilis* [88], the *E. coli* *ΔtcdA* strain displays impaired growth fitness when compared to the wild-type strain [196].

Methylthiolation of C2 on A37 is performed by a class of enzymes called methylthioadenosine transferases (MTTases). MTTases are comprised of three different families: MiaB, and MtaB, which modify tRNA A37, and RimO, which methylthiolates an aspartic acid residue on ribosomal protein S12 (enzyme families are represented here by their names in bacteria). Though not universally conserved, MiaB and MtaB homologs are dispersed between all three domains of life, whereas RimO is mainly found in eubacteria and some algae and fungi. All three MTTases contain a C-terminal TRAM domain (named after TRM2 and MiaB), typically involved in substrate recognition, and 2 [4Fe-4S] clusters. The first cluster, deemed the radical SAM cluster, is ligated by three conserved cysteine residues within a CXXXCXXC motif in the central domain of the protein, and the second (auxiliary) cluster is also ligated by three conserved cysteines in the N-terminal domain [197,198,199,200].

The requirement of the MiaB MTTase in methylthiolation at the C2 position of i^6^A, producing ms^2^i^6^A, was first reported in *E. coli* and *S. typhimurium*, but its homologs are found in both eukaryotes and eubacteria [33]. Archaeal species with fully sequenced genomes contain only one MTTase, with homology to cyclin-dependent-like kinase 5 repressor/activator site-binding protein 1-like 1 (CDKAL1) [201], the mammalian enzyme responsible for methylthiolation of ms^2^t^6^A [112]. Although the archaeal homologs have yet to be characterized experimentally, bioinformatics suggests that either archaea lack the ms^2^i^6^A37 modification or that the CDKAL1 homolog can act on both i^6^A and t^6^A substrates. The ms^2^i^6^A modification provides stability in interactions between tRNA and mRNA and the ribosome, and thus not surprisingly, defects in its biosynthetic enzyme MiaB result in translational frameshifting. In *S. typhimurium*, mutations within the *miaB* gene, rendering it inactive, demonstrated that absence of the ms^2^-modification on A37 reduced decoding efficiency of amber suppressor tRNA, and furthermore, affected regulation of several operons encoding components of amino acid biosynthesis [33].

Though present in *B. subtilis*, the MtaB (YqeV, TmtB) enzyme, and its product, the ms^2^t^6^A modification, are not found in *E. coli* [111,112]. Upon discovery of the role of MtaB in *B. subtilis* ms^2^t^6^A methylthiolation, the authors were interested in determining what governed the specificity of the MiaB and MtaB enzymes, as they catalyze strikingly similar reactions and exhibit a high degree of sequence similarity, yet simultaneously are specific for their respective i^6^A and t^6^A tRNA substrates. This prompted the generation of chimeric proteins in which the domains of MiaB and MtaB were switched, and subsequently tested for their competency to methylthiolate each other’s *N*^6^-modified tRNA substrates. These experiments, along with bioinformatic amino acid sequence analysis, led to the proposal that the radical SAM and/or N-terminal domains, but not the C-terminal TRAM domain were responsible for substrate recognition. In the same study, the authors also demonstrated that compared to its *E. coli* ortholog, *B. subtilis* MiaB is more restrictive in its i^6^A37-modified tRNA substrate recognition. *E. coli* MiaB is capable of modifying non-natural substrates such as an i^6^A37-containing 17 bp oligonucleotide mimicking the anticodon of a natural substrate, tRNA^Phe^, as well as several mature tRNAs mutated to become isopentylated by MiaA. Although comparison of tRNA substrate sequences across species enabled speculation as to the determinants of enzyme recognition, the difference in ms^2^i^6^A prevalence attributed to tRNA substrate sequence versus differences in the enzymes remains to be determined. Interestingly, ms^2^i^6^A abundance is associated with sporulation in *B. subtilis*, and considering the lack of this process in *E. coli*, has been hypothesized to be a source of divergence between the species [111]. Recent advances in techniques for analysis of modification levels, as well as precise location of modifications within tRNA isoacceptors will enable more thorough characterization of the enzymes involved in these pathways.

**Figure 5:**
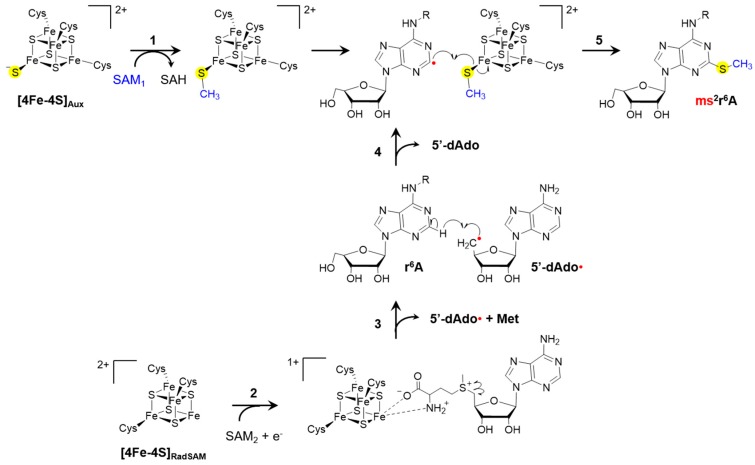
Proposed mechanism for methylthiolation of tRNA A37 by MiaB and MtaB. In the first step of the mechanism (1), a methyl group (in blue) is transferred from S-adenosylmethionine (SAM) to a sulfide ion (in yellow) previously ligated to the free Fe on the auxiliary [4Fe-4S] cluster. In steps 2 and 3, the radical SAM [4Fe-4S] cluster, via its free Fe, binds a second SAM molecule, which subsequently undergoes reductive cleavage, releasing methionine and the 5′-deoxyadenosyl radical (5′-dAdo•, in red). Next (4), the highly reactive 5′-dAdo• abstracts a H from C2 of tRNA r^6^A37 (previously modified at the C6 position), generating a r^6^A37• radical at C2 and releasing 5′-deoxyadenosine (5′-dAdo). In the final step (5), the r^6^A37• radical facilitates transfer of the methylthio group from the auxiliary 4Fe-4S to C2 of r^6^A37, yielding the final ms^2^r^6^A37 product. In this legend, “free Fe” refers to the only Fe within the cluster not ligated to a Cys residue within the enzyme.

Initial studies involving methylthiolation by MTTases showed inefficiency of these enzymes in catalyzing multiple turnover reactions. Based on the analysis of cofactors before and after reaction cycles, the mechanism for tRNA A37 methylthiolation was postulated to involve destruction (i.e., sacrifice) of the MTTase’s auxiliary cluster to provide the sulfur atom for thiolation of A37, followed by methylation of that sulfur in a polar S_N_2 reaction. This mechanism is consistent with that utilized by the radical SAM enzymes LipA and BioB in biosynthesis of lipoic acid and biotin, respectively [202,203]. Although the aforementioned mechanism was supported by experimental evidence [16,17,109,204], and similar to that of related enzymes, the inherent instability of [Fe-S] cluster in radical SAM enzymes may have perhaps confounded results by indicating a sacrificial role for the auxiliary [Fe-S] cluster in these MTTases. Recent studies have provided evidence for an alternate distinct mechanism in which the sulfur is not obtained from the [4Fe-4S] cluster itself, but rather a sulfide ion previously ligated to the only Fe atom on the auxiliary cluster that is not coordinated by a Cys residue (Figure 5). Although the source of the sulfide ion is as yet unknown, the presumption is that it is obtained from free cysteine.

There are two major branches for the ms^2^A37 mechanism, each involving one of the MTTase clusters. First, the sulfide ion ligated to the auxiliary cluster becomes methylated in a reaction using SAM and resulting in s-adenosylhomocysteine(SAH) as a byproduct. Meanwhile, the “free” Fe on the radical SAM cluster is coordinated by a second molecule of SAM, and reductive cleavage of this SAM molecule results in release of methionine and more importantly, the 5′-deoxyadenosyl radical, which can abstract an H from C2 of r^6^A37 (i^6^-, t^6^-, and ct^6^-modifications are represented here and in Figure 5 as r^6^- for simplicity), yielding a r^6^A37 radical. Finally, the two branches are joined together, as the r^6^A37 radical enables transfer of the methylthio group from the auxiliary cluster to its C2 position [13,14,15,200,205].

## 4. Interconnectivity between tRNA Modification and Biosynthesis of Other Cofactors

Unlike the thio-modifications that are conserved throughout all kingdoms, it appears that some thio-modifications are not necessary in eukaryotes. For instance, s^2^C is only observed in bacterial tRNA^Ser^ and tRNA^Arg^ [63] and it is absent in several species of bacteria including the model Gram-positive bacterium *B. subtilis*. Furthermore, s^4^U is likely the most abundant thionucleoside in prokaryotic tRNA [62], present in 25 of the 40 tRNA isoacceptors in *E. coli* [3], yet it is absent in eukaryotes. These modifications are not strictly required for cellular growth, but rather, impact downstream translational events by altering tRNA structural stability. In particular, the thiol modification from cytosine at position 32 enhances the interaction between the first (C32) and last (A38) nucleotides in the anticodon loop and results in optimal decoding efficiency [63]. s^4^U’s photochemical reaction, as discussed above, leads to disordered tRNA structure and reduces the cell growth rate by triggering the stringent response [62]. Recent studies have revealed that thio-modifications participate in other cellular processes, including metabolism, stress response, and regulatory functions [24]. Several recent models have proposed interconnectivity between biosynthesis of thionucleosides and other thiocofactors in bacteria, providing another potential regulatory mechanism for thiocofactor biosynthesis [178,179]. In *E. coli*, s^2^C and ms^2^i^6^A biosynthetic pathways utilize [Fe-S] cluster dependent proteins, as do the pathways for ms^2^i^6^A and ms^2^t^6^A biosyntheses in *B. subtilis*. It is likely that levels of such modifications are linked to the competency of [Fe-S] cluster biogenesis (Table 1), which is essential for bacterial survival and sensitive to environmental stresses. In addition, a non-thiolation modification, queuosine at the tRNA (His, Asp, Asn, Glu and Tyr) wobble position is synthesized in 8 enzymatic steps, with the final step catalyzed by the [Fe-S] cluster and cobalamin-dependent enzyme QueG [103]. This modification has become an element to assess the global biogenesis of [Fe-S] clusters. Although s^4^U and xm^5^s^2^U formation are not dependent on [Fe-S] clusters, their sulfur mobilization pathways are also shared with those of thiamine and molybdenum cofactor biosyntheses, respectively. Thus, deficiencies in these two tRNA modifications usually coincide with depletion of thiamine and/or molybdenum cofactor simultaneously [11]. An interesting implication of the model for thiocofactor interconnectivity in *E. coli* is the potential for a hierarchical order of sulfur delivery to various thiocofactors, mediated by competition between sulfur acceptor proteins for interaction with the cysteine desulfurase, IscS. This competition could be affected by many variables, including but not limited to, the binding affinity of IscS for its various sulfur acceptor proteins, overlapping regions of interaction on IscS, and expression levels of the sulfur acceptor protein substrates. Similarly, sulfur flux to various pathways could also be regulated by expression levels of downstream pathway components, like MnmA for s^2^U formation, or the final tRNA substrate itself. In contrast, *B. subtilis* has adopted an alternative strategy by encoding several cysteine desulfurases with devoted functions in sulfur mobilization. The mechanism for regulating sulfur incorporation into tRNA is partially decoupled from the biosynthesis of other sulfur cofactors in this Gram-positive bacterium.

Although recent work has allowed us to take significant leaps in our understanding of bacterial thiocofactor formation, several additional questions remain regarding thionucleoside biosynthesis in both systems. Though the mechanism for uridine adenylation and thiolation has been proposed, the exact residues initiating adenylation are still unclear for ThiI and MnmA. The dependency of ATP-binding for the catalytic sulfurtransferase competency of MnmA, whether structural or otherwise, which is dispensable for ThiI, has yet to be determined. In both MnmA and ThiI reaction mechanisms, the end of each thiouridylase’s catalytic cycle is marked with the formation of a disulfide bond, enforcing the requirement of a reductive step for the regeneration of the enzyme. This reaction scheme poses a question regarding the identity of the physiological reducing agent participating in these biosynthetic pathways. Equally fascinating, and yet not fully understood, are the structural and mechanistic determinants of the MTTase enzymes and their tRNA substrates governing specificity in tRNA modification reactions. Likewise, the identity of the sulfur source for A37 modifications and reaction schemes allowing for enzyme regeneration upon multiple turnovers still represent challenges when uncovering details about these pathways. Lastly, the capacity of involvement of the cysteine desulfurase and sulfur transfer enzymes, CsdA and CsdE, in formation of the non-sulfur containing ct^6^A modification still remains enigmatic.

Another emerging and exciting aspect in this area of study besides complex and interconnected biosynthetic pathways, is the identification of additional functions of tRNA in signaling metabolic and environmental changes and their consequent roles in regulating translation. The discovery of new roles for tRNA in cellular processes is greatly facilitated by the development of systems-levels approaches for assessing tRNA modification fluctuations in response to various stress and growth conditions, which was first utilized in yeast [41]. A more recent application of the systems-level approach by the same group revealed that a similar phenomenon occurs in *Mycobacterium bovis* BCG, which reprograms 40 modified nucleosides in response to hypoxia, consequently affecting translation of various genes involved in bacterial persistence [151]. A detailed investigation was conducted to understand the nearly 8-fold increase in cmo^5^U observed upon exposure to hypoxia, but this experiment also revealed a significant change in thionucleosides that has yet to be explored. Importantly, ms^2^i^6^A levels increased >5-fold in response to hypoxia, with a corresponding depletion of i^6^A, whereas ms^2^t^6^A was completely depleted during hypoxia and a concomitant 2-fold increase in t^6^A and ct^6^A was observed. U34 thiolation levels changed drastically in response to hypoxia as well, with a 4- and 10.6-fold increase in s^2^Um and mnm^5^s^2^U, respectively. Interestingly, hypoxia induced a depletion in cmnm^5^s^2^U, in addition to cmnm^5^U and mnm^5^U, suggesting that neither branch of U34 modification is impacted by hypoxia, particularly the thiolation branch. Alternatively, thiolation of s^4^U was negatively impacted during hypoxia, as it was completely depleted. For all examples given above, the levels of modified nucleosides returned back to normal levels upon reaeration, albeit some more quickly than others. Collectively, these results indicate that such methodology can potentially enable predictions of proteins that are required during hypoxia and bacterial persistence by means of identifying genes enriched in codons read by tRNAs containing modifications that are upregulated during these conditions. Similarly, it could suggest mechanisms for exiting persistence, based on tRNA modifications that are depleted during hypoxia, yet increase drastically within the first stage of reaeration.

Recent technological advances on methods for detection and quantification of relative levels of tRNA modifications propelled by the rapid expansion of the inventory of bacterial species with sequenced genomes has provided valuable tools for uncovering new functions of sulfur decorations on tRNA. Post-transcriptional modifications on tRNA are not only important in guaranteeing the accurate decoding of genetic information but also connecting the universal process of translation to other metabolic pathways involving the synthesis of protein cofactors as well as cellular adaptations to environmental and nutritional changes.

## Figures and Tables

**Table 1:** Bacterial tRNA modifications whose biosynthesis relies on sulfur mobilization.

Fe-S Cluster Independent Modifications				
Modification	Name		Position	Biosynthetic Genes and Precursors
s^4^U	4-thiouridine		8	*iscS-thiI* (*Escherichia* *coli* [11,62,64,65], *Salmonella enterica* [66] and *Thermatoga maritima* [67]); or *nifZ-thiI* (*Bacillus subtilis* [68] and *Bacillus anthracis* [69])
s^2^U	2-thiouridine		34	*iscS-tusABCDE-mnmA* (*E. coli* [11,37,70,71], *S. enterica*, [52]); or *yrvO-mnmA* (*B. subtilis* [72])
mnm^5^s^2^U	5-methylaminomethyl-2-thiouridine		34	s^2^U, *mnmEG/gidA*, *mnmC1-2* (*E. coli* [73]); or s^2^U, *mnmEG/gidA*, *mnmC2* (*Aquifex aeolicus* [74])
cmnm^5^s^2^U	5-carboxymethylaminomethyl-2-thiouridine		34	s^2^U, *mnmEG/gidA* (*E. coli* [75,76,77,78])
inm^5^s^2^U	5-(isopentenylaminomethyl)-2-thiouridine		34	nm^5^s^2^U, unknown isopentenyltransferase (*Thermodesulfobacterium commune*)
nm^5^s^2^U	5-aminomethyl-2-thiouridine		34	s^2^U, *mnmEG/gidA*, *mnmC1* (*E. coli* [73])
se^2^U	2-selenouridine		34	s^2^U, *selU/mnmH, selD* (*S. enterica* [79] and *E. coli* [80])
mnm^5^se^2^U	5-methylaminomethyl-2-selenouridine		34	mnm^5^s^2^U, *selU/mnmH, selD* (*S. enterica* [79,81,82], *E. coli* [80])
ges^2^U	2-geranylthiouridine		34	s^2^U, *selU/mnmH* (*E. coli* [83])
cmnm^5^ges^2^U	5-carboxymethylaminomethyl-2-geranylthiouridine		34	cmnm^5^s^2^U, *selU/mnmH* (*S. enterica* [29,81], *E. coli* [29,83], *Enterobacter aerogenes*, and *Pseudomonas aeruginosa* [29])
mnm^5^ges^2^U	5-methylaminomethy-2-geranylthiouridine		34	mnm^5^s^2^U, *selU/mnmH* (*S. enterica* [29,81], *E. coli* [29,83], *E. aerogenes* and *P. aeruginosa* [29])
nm^5^ges^2^U	5-aminomethyl-2-geranylthiouridine		34	nm^5^s^2^U, proposed *selU/mnmH* (*S. enterica* and *E. coli*)
ct^6^A	cyclic *N*^6^-threonylcarbamoyladenosine		37	t^6^A*, tcdA/csdL* (*E. coli* [59,84,85,86,87], *B. subtilis* [88])
m^5^s^2^U/s^2^T	5-methyl-2-thiouridine/2-thioribothymidine		54	*ttuBCA* (*Thermus thermophilus* [10,89,90,91,92], *Pyrococcus furiosus* [93])
**Fe-S Cluster Dependent Modifications**				
**Modification**		**Name**	**Position**	**Biosynthetic Genes and Precursors**
s^2^C	2-thiocytidine		32	*ttcA* (*E. coli* [63,94])
Q	queuosine		34	*folE, queACDEFG, tgt,* (*E. coli* [95,96,97,98,99,100,101,102], *S. enterica* [95], *B. subtilis* [97,98,103,104], *Streptomyces rimosus* [105], *Acinetobacter* sp. and *Zymomonas mobilis* [106])
m^2^A	2-methyladenosine		37	*trmG/rlmN* (*E. coli* [107,108])
ms^2^i^6^A	2-methylthio-*N*^6^-isopentenyladenosine		37	i^6^A, *miaB* (*T. maritima* [13,15,16,17], *E. coli* [33,109], *S. enterica* [33], *Shigella flexneri* [110] and *B. subtilis* [111,112])
ms^2^io^6^A	2-methylthio-*cis*-ribozeatin		37	ms^2^i^6^A, *miaE* (*S. enterica* [113,114])
ms^2^t^6^A	2-methylthio-*N*^6^-threonyl carbamoyladenosine		37	t^6^A, *yqeV/mtaB* (*B. subtilis* [111,112])
ms^2^ct^6^A	2-methylthio-cyclic-*N*^6^-threonyl carbamoyladenosine		37	ms^2^t^6^A, *tcdA/csdL* (*B. subtilis* [88])

**Table 2:** General methods for detection and analysis of tRNA modifications.

Method	Target Modification	Advantage	Disadvantage
Chemical labeling	s^2^U, s^4^U, mnm^5^s^2^U, pseudouridine	Labeling only occurs in the modified species; high selectivity	Detection method varies with modification and potential side reactions
Northern Blot	all	High sensitivity towards specific tRNA sequences	Unable to differentiate modified and canonical nucleotides; requires RNA probe for each cognate tRNA; may require radioactive probe
Immuno-Northern Blot	m^1^A, m^6^A, m^5^C	Antibodies bind specifically to the modified nucleoside	Limited antibodies
APM-gel	s^4^U, s^2^U and derivatives	Detect polynucleotides and single nucleosides; simple analysis	Hazardous mercury compound involved; varied sensitivity toward different thionucleotides
APB-gel	Q	Specific to Q modification	Reactive with *cis*-diol functional groups
HPLC-UV/vis	all	Simple sample preparation and data quantification. Certain nucleosides have unique absorbance λ max	Nuclease and phosphatase treatments
HPLC-MS	all	High sensitivity and accuracy	Detection may result of fragmentation of certain modifications

APB: *N*-acryloyl-3-aminophenylboronic acid; APM: [(*N*-acryloylamino)phenyl]mercuric chloride; HPLC-UV: High Pressure Liquid Chromatography separation coupled to ultraviolet detection; HPLC-MS: High Pressure Liquid Chromatography separation coupled to Mass Spectrometry detection.

**Table 3:** Molecular masses of tRNA modifications dependent on sulfur metabolism and standards.

S-Dependent Modifications					
Modification	Name	Monoisotopic Mass (amu)	Observed Molecular Ion (*m/z+*)	Major Fragment (*m/z+*)	Standard **
s^2^C	2-thiocytidine	259.063	260.071	128.028	1
s^2^U	2-thiouridine	260.047	261.055	129.009 *	1
s^4^U	4-thiouridine	260.047	261.055	129.009 *	2
s^2^Um	2-thio-2′-*O*-methyluridine	274.062	275.067	129.008 *	
m^5^s^2^U/s^2^T	5-methyl-2-thiouridine/2-thioribothymidine	274.062	275.062	143.019	1, 2
m^2^A ^†^	*N*^2^-methyladenosine ^†^	281.112	282.114	150.071	
nm^5^s^2^U	5-methylaminomethyl-2-thiouridine	289.073	290.081	158.038	
mnm^5^s^2^U	5-methylaminomethyl-2-thiouridine	303.089	304.097	172.057 *	
se^2^U	2-selenouridine	305.992	306.999	174.956	
ms^2^m^6^A	2-methylthio-*N*^6^-methyladenosine	327.100	328.108	196.065	
cmnm^5^s^2^U	5-carboxymethylaminomethyl-2-thiouridine	347.079	348.082	216.047 *	
mnm^5^se^2^U	5-methylaminomethyl-2-selenouridine	349.034	350.034	217.991	
inm^5^s^2^U	5-(isopentenylaminomethyl)-2-thiouridine	357.136	358.144	226.101	
ms^2^i^6^A	2-methylthio-*N*^6^-isopentenyladenosine	381.147	382.155	250.108 *	1
cmnm^5^se^2^U	5-carboxymethylaminomethyl-2-selenouridine	393.024	394.032	261.989	
ct^6^A	cyclic *N*^6^-threonylcarbamoyladenosine	394.124	395.128	263.089 *	
ges^2^U	2-geranylthiouridine	396.172	397.180	265.137	
ms^2^io^6^A	2-methylthio-*N*^6^-(*cis*-hydroxyisopentenyl) adenosine	397.142	398.150	266.107	
Q	queuosine	409.160	410.168	278.125	
nm^5^ges^2^U	5-methylaminomethyl-2-geranylthiouridine	425.198	426.206	294.163	
mnm^5^ges^2^U	5-methylaminomethyl-2-geranylthiouridine	439.214	440.222	308.179	
ms^2^ct^6^A	2-methylthio-cyclic-*N*^6^-threonyl carbamoyladenosine	440.111	441.119	309.076	
ms^2^t^6^A	2-methylthio-*N*^6^-threonyl carbamoyladenosine	458.112	459.130	182.049 *	
ms^2^hn^6^A	2-methylthio-*N*^6^-hydroxynorvalylcarbamoyladenosine	472.138	473.146	341.103	
cmnm^5^ges^2^U	5-carboxymethylaminomethyl-2-geranylthiouridine	483.204	484.212	352.169	
**Precursor and Related Modifications**					
**Modification**	**Name**	**Monoisotopic Mass (amu)**	**Observed Molecular Ion (*m/z+*)**	**Major Fragment (*m/z+*)**	Standard **
cmnm^5^Um	5-carboxymethylaminomethyl-2′-*O*-methyluridine	345.117	346.117	200.058 *	
m^6^A	*N*^6^-methyladenosine ^†,‡^	281.112	282.114	150.071 *	1
mnm^5^U	5-methylaminomethyluridine	287.112	288.120	156.074 *	
i^6^A	*N*^6^-isopentenyladenosine	335.159	336.167	204.119 *	1
inm^5^U	5-(isopentenylaminomethyl) uridine	341.159	342.167	210.135	
io^6^A	*N*^6^-(*cis*-hydroxyisopentenyl) adenosine	351.154	352.156	220.115 *	
inm^5^Um	5-(isopentenylaminomethyl)-2′-*O*-methyluridine	355.174	356.182	224.139	
t^6^A	*N*^6^-threonylcarbamoyladenosine	412.134	413.142	136.062 *	3
oQ	epoxyqueuosine	425.155	426.163	294.120	
hn^6^A	*N*^6^-hydroxynorvalylcarbamoyl adenosine	426.150	427.158	295.115	
**Common Internal Standards**					
**Modification**	**Name**	**Monoisotopic Mass (amu)**	**Observed Molecular Ion (*m/z+*)**	**Major Fragment (*m/z+*)**	Standard **
I	Inosine ^‡^	268.081	269.088	137.047 *	2
Ψ	Pseudouridine ^†^	244.070	245.078	209.052 *	2
D	Dihydrouridine ^†^	246.085	247.092	115.050 *	4, 5

* major fragment masses have been experimentally validated [151]; ** commercially available standards: 1-Carbosynth Ltd., Berkshire, United Kingdom, 2-Sigma-Aldrich, St Louis, MO, USA, 3-BioLog Life Science Institute, Bremen, Germany, 4-Advanced Technology & Industrial Co., Ltd., Hong Kong, China, 5-Dalton Pharma Services, Toronto, Canada; ^†^ Also found in bacterial ribosomal RNA; ^‡^ Has not been found in bacterial messenger RNA, but occurs in eukaryotic mRNA.
